# Effect of rear impact on the instrumented cervical spine: a finite element study

**DOI:** 10.3389/fbioe.2025.1681077

**Published:** 2026-01-07

**Authors:** Balaji Harinathan, Davidson Jebaseelan, Narayan Yoganandan

**Affiliations:** 1 Department of Neurosurgery, Medical College of Wisconsin, Milwaukee, WI, United States; 2 School of Mechanical Engineering, Vellore Institute of Technology, Chennai, India

**Keywords:** cervical spine, spinal cord compression, spinal cord injury, finite element model, spinal cord strain, rear impact

## Abstract

**Introduction:**

Degenerative cervical myelopathy (DCM) is a leading cause of spinal cord dysfunction and often requires surgical decompression and instrumentation. Anterior cervical discectomy and fusion (ACDF) is the most commonly performed procedure, but the biomechanical response of instrumented cervical spines under rear-impact loading remains unclear.

**Methods:**

A validated finite element model of the head–neck complex with active musculature, spinal cord, and three instrumented constructs was used to simulate a low-speed rear impact corresponding to a 2.6 m/s change in velocity. The following surgical conditions were modeled at C4–C7: ACDF, posterior cervical laminectomy and fusion (PCLF), and laminoplasty (LP). Spinal cord stress and strain, ligament strain (including anterior longitudinal and capsular ligaments), and implant stress were evaluated at the index, superior, and inferior segments.

**Results:**

Compared to ACDF, both PCLF and LP reduced spinal cord stress and strain at the index level, with PCLF showing the greatest reduction. LP preserved motion but increased facet capsular ligament strain at the index and adjacent levels. Anterior longitudinal ligament strains remained below 36%, within the reported 40–45% failure range, while PCLF screws exceeded the 900 MPa yield strength of Ti–6Al–4V at all levels except C4, indicating a reduced hardware safety margin. ACDF and LP implants remained within safe stress limits.

**Conclusion:**

Under rear-impact loading, PCLF reduced spinal cord stress and strain relative to ACDF but increased hardware stresses, whereas LP preserved motion at the cost of higher facet capsular ligament strain. These findings suggest that PCLF may be preferred when minimizing spinal cord loading is a priority, while LP may be selected when motion preservation is critical, with awareness of construct-specific trade-offs in ligament and implant loading.

## Introduction

Degenerative cervical myelopathy (DCM) is the leading cause of spinal cord dysfunction in adults, resulting from chronic cervical spinal cord compression due to degenerative changes (Smith et al., 2021). This condition significantly impacts public health, with its progressive nature reducing quality of life. Epidemiological data indicate an increasing prevalence, with reporting that 62% of individuals over 40 exhibit degenerative cervical changes, underscoring the need for effective management. Traffic accidents further exacerbate this burden, contributing to over 1.25 million global deaths and 20–50 million non-fatal injuries annually, including a substantial number of cervical spine traumas (World Health Organization, 2023). Neck injuries account for 66% of occupant trauma in automotive collisions, demonstrating the intersection between DCM and post-operative vulnerabilities (Guo et al., 2024).

Surgical decompression with instrumentation remains the primary intervention for DCM, aiming to restore mechanical stability and alleviate symptoms. ACDF is the most frequently performed procedure, achieving fusion rates exceeding 95%. In the United States, approximately 150,000 cervical spine surgeries are performed annually using the ACDF, compared to 30,000 using the posterior approach (Epstein, 2019). The number of anterior cervical surgeries is projected to increase by 13% between 2020 and 2040, while posterior cervical procedures are expected to rise by 19% in response to an aging population and increasing case complexity (Mohamad Radzi et al., 2021). Despite the efficacy of these interventions, rigid instrumentation alters spinal biomechanics, potentially increasing vulnerability under dynamic loading conditions such as automotive impacts.

Rear impact collisions account for 57.7% of global car accident cases (Razzak et al., 2022). Whiplash injuries, although classified as minor trauma, can result in capsular ligament damage, anterior longitudinal ligament tears, disc fiber disruption, and direct spinal cord trauma (Panjabi et al., 1998). Guo et al. (2024) report that 50% of neck injury come from automotive crashes, with frontal impacts alone causing severe spinal fractures in 5,592 ± 1,170 cases annually (Guo et al., 2024; Parenteau and Viano, 2014). Patients with prior cervical instrumentation may experience catastrophic secondary injuries, yet the biomechanical response of instrumented spine to rear impacts remains unclear. White et al. (2016) compared arthroplasty and fusion in frontal impacts, omitting spinal cord injury (White et al., 2016). Huang et al. (2018) analyzed anterior longitudinal ligament strain post-fusion during rear impact but did not assess posterior approaches or spinal cord injury (Huang et al., 2018). FE model have been enhanced the understanding of cervical spine biomechanics under automotive impact; however, most studies evaluated non-instrumented spines, the limited instrumented spine models did not include the spinal cord. Guo et al. (2024) evaluated ACDF under frontal impacts, increased vertebral destruction with additional fusion levels but did not investigate rear impact conditions or alternative surgical techniques such as PCLF and LP (Guo et al., 2024). The absence of comprehensive analyses examining instrumented spines, including spinal cord, under rear impact loading limits understanding of post-operative trauma risks and stability of cervical spine. Under dynamic rear-impact loading, muscle activation can reduce capsular ligament (CL) strain from 28% to 13% in 7 g simulations (Fice et al., 2011). Facet joints are a primary source of chronic post-whiplash neck pain in about 60% of cases, and cadaver studies have reported CL strains exceeding a sub-failure threshold of 35% at 5 g and 10.5 g (Panjabi et al., 1998; Panjabi et al., 2004; Lord et al., 1996). For the anterior longitudinal ligament (ALL), failure-related strain thresholds of 40%–45% have been reported (Henderson et al., 2005). Instrumented constructs are evaluated by comparing implant stresses to the 900 MPa yield strength of Ti-6Al-4V (Kulkarni et al., 2024; Tadepalli and Grosland, 2025).

This study addresses these gaps by utilizing a validated FE model of the head-neck, incorporating active musculature, disc, ligaments, and the spinal cord, to compare biomechanical outcomes of ACDF, PCLF, and LP following rear impact trauma. A low-speed rear impact (2.6 m/s velocity change) is simulated to quantify spinal cord stress, ligament strain, and instrumentation stress across these surgical interventions. The findings provide critical data on the stability and injury risks of surgically altered cervical spines, informing surgical decision-making and occupant protection strategies in prevalent rear impact trauma.

## Materials and methods

**FIGURE 1:**
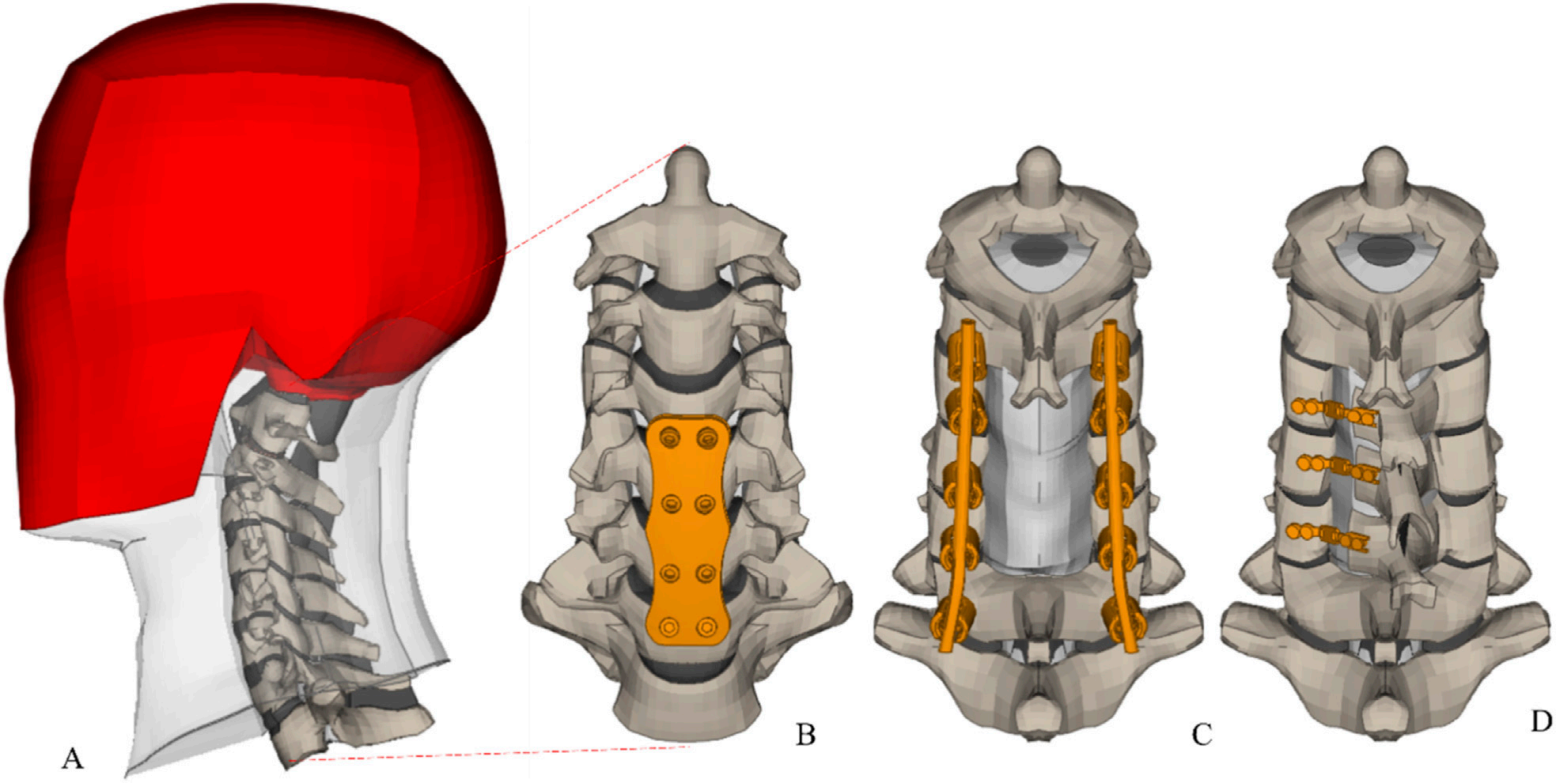
**(A)** FE model of head and neck **(B)** ACDF at C4-C7 **(C)** PCLF at C4-C6 and fusion C3-C7 **(D)** LP at C4-C6.

**FIGURE 2:**
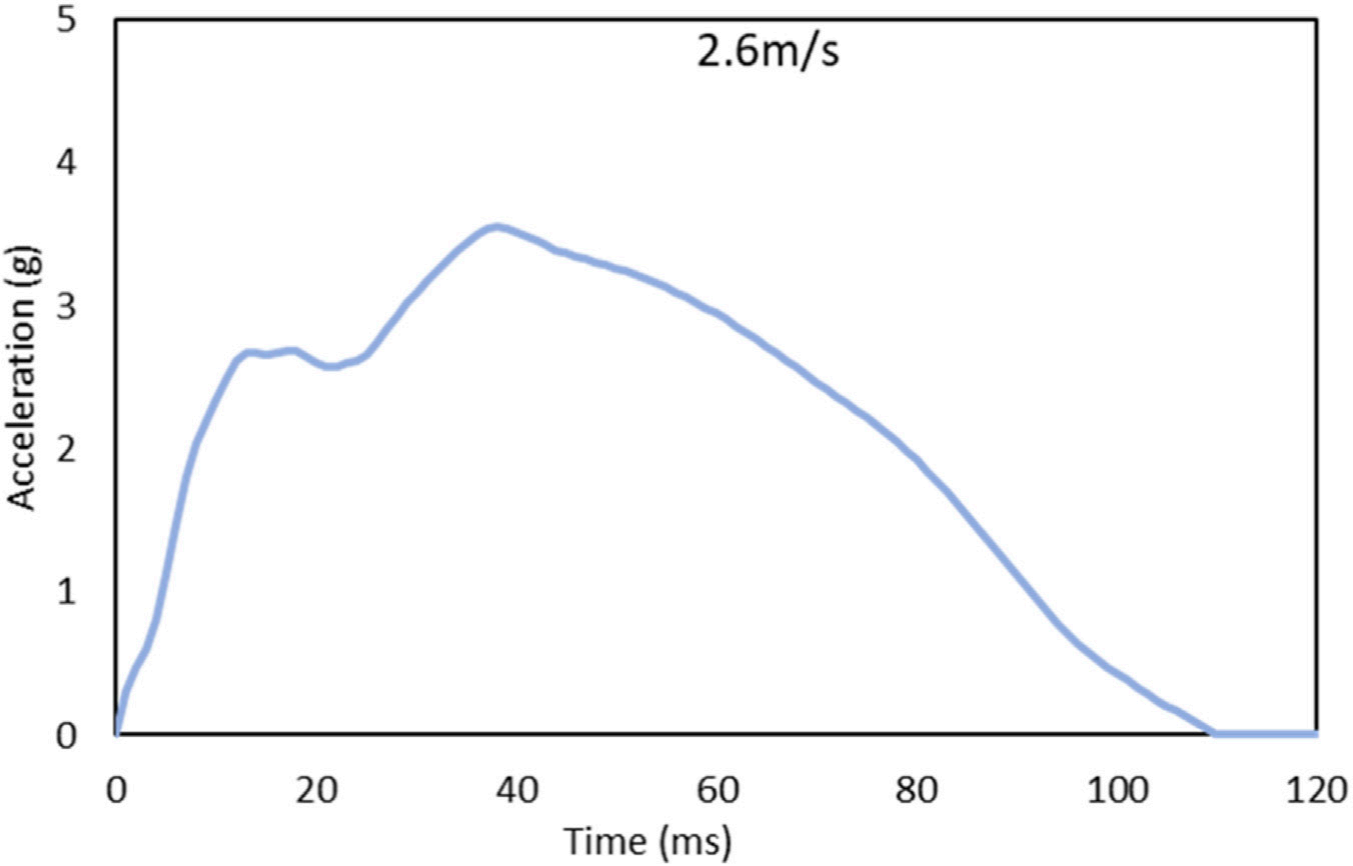
The acceleration time‐history (2.6 m/s, 120 ms).

We used our validated 3D head-neck FE model (John et al., 2019). This model is developed using a multi-block hexahedral meshing technique (John JD. et al., 2017). It encompasses the osteoligamentous cervical spine column from C1 to T1 (Figure 1A), integrating vertebral bodies, posterior elements, intervertebral disks, facet joints with their cartilage, and ligaments. The subaxial cervical spinal column model also featured the spinal cord, active musculature, and a rigid head (Vedantam et al., 2023a). The vertebral components are detailed with layers representing cortical and cancellous bone, topped and bottomed by superior and inferior endplates. For intervertebral disks, we distinguished between annulus and nucleus pulposus, each modeled with specific elements to capture their unique material properties (Jobin JD. et al., 2017). The annulus fibrosus represented by quadratic shell elements, reflecting its nonlinear behavior, while the nucleus pulposus is modeled with solid hexahedral elements. This model is comprehensive, featuring all critical ligaments, the spinal cord, and cervical muscles using the Hill-type muscle model. This includes representation of 23 pairs of cervical spine muscles, with placement informed by FE study (John et al., 2019). We also include muscle activation through the active Hill-type muscle, contributing to a dynamic understanding of muscular interactions within the cervical spine (Harinathan et al., 2023). The acceleration time‐history (2.6 m/s over 120 ms) shown in Figure 2. Muscle activation for flexors muscle group is activated from 74 ms to 120 ms after impact, and extensor muscle group activation is set to 70% of the flexor level. The spinal cord-spinal canal interface as frictionless surface-to-surface contact, representing the lubricated interface created by cerebrospinal fluid. Table 1 Contains material properties of the cervical spine, spinal cord and surgical construct components.

**TABLE 1:** Material properties of the cervical spine, spinal cord and surgical construct components.

Component	Element type	Constitutive model	Values for the material model	References
Cortical bone	Quadrilateral shell	Linear elastic	E = 16.8 GPa, n = 0.3	Parenteau and Viano (2014)
Trabecular bone	Hexahedral solid	Linear elastic	E = 0.442 GPa, n = 0.3	White et al. (2016)
Endplates	Quadrilateral shell	Linear elastic	E = 5.6 GPa, n = 0.3	Huang et al. (2018)
Annulus ground substance	Hexahedral solid	Hill Foam	C_1_ = 0.000115 GPa, C_2_ = 0.002101 GPa, C_3_ = −0.000893 GPa, b_1_ = 4, b_2_ = −1, b_3_ = −2	Guo et al. (2024)
Annulus fibrosus	Quadrilateral membrane	Orthotropic nonlinear	Fiber angle: 45–60°	Fice et al. (2011), Panjabi et al. (2004)
Nucleus pulposus	Hexahedral solid	Fluid	K = 1.720 GPa	Guo et al. (2024)
Ligaments	Quadrilateral membrane	Non-linear	Stress-Strain curves	Lord et al. (1996)
Spinal cord	Hexahedral solid	Viscoelastic	ρ = 1.04e3 kg/m^3^, C1 = 0.5345, C2 = 1.0665, C3 = 1.0113, G1 = 0.8927, G2 = 0.8926, G3 = 0.8917, β1 = −0.0137, β2 = 0.00775, β3 = 0.035	Kulkarni et al. (2024)
Pia mater	Quadrilateral shell	Linear Elastic	ρ = 1.13e3 kg/m^3^, *E* = 2.3 MPa, *ν* = 0.49	Tadepalli and Grosland (2025)
Dura mater	Quadrilateral shell	Linear Elastic	ρ = 1.130e3 kg/m^3^, *E* = 80 MPa, *ν* = 0.49	John et al. (2019)
Denticulate ligaments	Quadrilateral shell	Linear Elastic	ρ = 1.040e3 kg/m^3^, *E* = 0.0058 GPa, *ν* = 0.45	John et al. (2017a)
Cerebral spinal fluid	Hexahedral solid	Viscoelastic	ρ = 1.040e3 kg/m^3^, K = 2.19 GPa, G0 = 5e-7, G1 = 1e-7	Vedantam et al. (2023a)
Surgical construct	Plate/rod	Linear elastic	E = 110 GPa, µ = 0.3	John et al. (2017b)
	Screw	Linear elastic	E = 110 GPa, µ = 0.3	
	ACDF-graft	Linear elastic	E = 0.45 GPa, µ = 0.29	

The ACDF FE modelling mimics the surgical insertion of a graft between the C4-C5, C5-C6, and C6-C7 vertebral bodies, aligning with the procedural norms. Utilizing CATIA V6 software (Dassault systems, USA). Figure 1B shows, 3D models of the anterior cervical plate and its screws, which have variable angles. These screws, standardized to a length of 18 mm and a diameter of 3 mm, were integrated into our FE model (Vedantam et al., 2023a). To ensure the graft’s stability, constraints mimicking the surgical attachment to the adjacent vertebral bodies were applied. The material properties of the surgical instruments were consistent with current research findings. Contact was simulated by using a ‘tied’ constraint for the screw-vertebra and an automatic surface-to-surface contact for the screw-plate and plate-vertebra. The ACDF’s biomechanical impact was demonstrated by modeling a 1 mm ventral CSF column at the decompressed sites, reflecting the surgical outcome.

In the PCLF simulation, excision was performed on the C5-C6 segment’s spinous process, lamina, and ligamentum flavum, extending to adjacent segments. Using CATIA V6, (Dassault systems, USA). We simulated the fusion from C4 to C7 (Figure 1C), incorporating a custom-modeled titanium rod with a variable curvature to align with the lateral mass screws. Contact was simulated by using a ‘tied’ constraint for the screw-vertebra, screw-rod and an automatic surface-to-surface contact for the rod-vertebra (Vedantam et al., 2023b). Post-surgical biomechanical changes were represented by a dorsal shift of the spinal cord by 2 mm at the treated segments, mirroring the expected post-decompression displacement (Ashana et al., 2021).

For the open-door LP model, a full-thickness trough was created on one side of the lamina at C4–C6, and a partial-thickness trough was created on the contralateral side to function as a hinge (Figure 1D). Following the lamina and ligament modifications, the laminae were rotated towards the hinge, simulating the surgical procedure (Vedantam et al., 2023b). The implementation of a titanium plate, meshed using hexahedral elements through ANSA software (BETA CAE systems, USA), secured the LP, Contact was simulated by using a ‘tied’ constraint for the screw-vertebra and an automatic surface-to-surface contact for the plate-vertebra (Vedantam et al., 2023b).

Our model underwent a rear impact simulation, where linear acceleration was applied to the T1 vertebrae, inducing a velocity change of 2.6 m/s (5.82 mph) in the Gx direction (Figure 2), aligning with real-world data derived from cadaver experiments in our facility (Stemper et al., 2004). The model incorporated a 4.06 kg head mass, represented as a point mass. Head positioning matched the experimental setup, with the Frankfort plane horizontal and the occipital condyles directly above T1. Passive and active muscle structures were included in the model, the applied acceleration pulse spanning 120 ms to capture the dynamic response. The FE analyses were conducted using the LS DYNA R10.0.0 solver, MATLAB for postprocessing simulations.

We quantitatively assessed key parameters to understand the biomechanics of an instrumented spine subjected to a low-speed rear impact. Specifically, we measured spinal cord stress and strain, range of motion, capsular ligament strain, anterior longitudinal ligament strain and instrument stress. Interventions included both anterior (ACDF) and posterior (PCLF, LP) approaches, and these metrics were compared with the ACDF The index level, which averages the operated levels (C4-C5, C5-C6, C6-C7); the superior level (C3-C4); and the inferior level (C6-C7).

## Results

Intact head-neck model was validated using range of motions derived from human cadaver experiments, focusing on segments ranging from C2–C3 to C6–C7. This validation process confirmed that the FE model response to loading aligned with experimental data (John et al., 2019; Purushothaman and Yoganandan, 2022). In the comparative analysis of biomechanical outcomes following ACDF, PCLF, and LP, we assessed the range of motion (ROM) at three critical spinal levels: superior, index, and inferior. The index level represents the average outcomes across the three decompressed regions.

**FIGURE 3:**
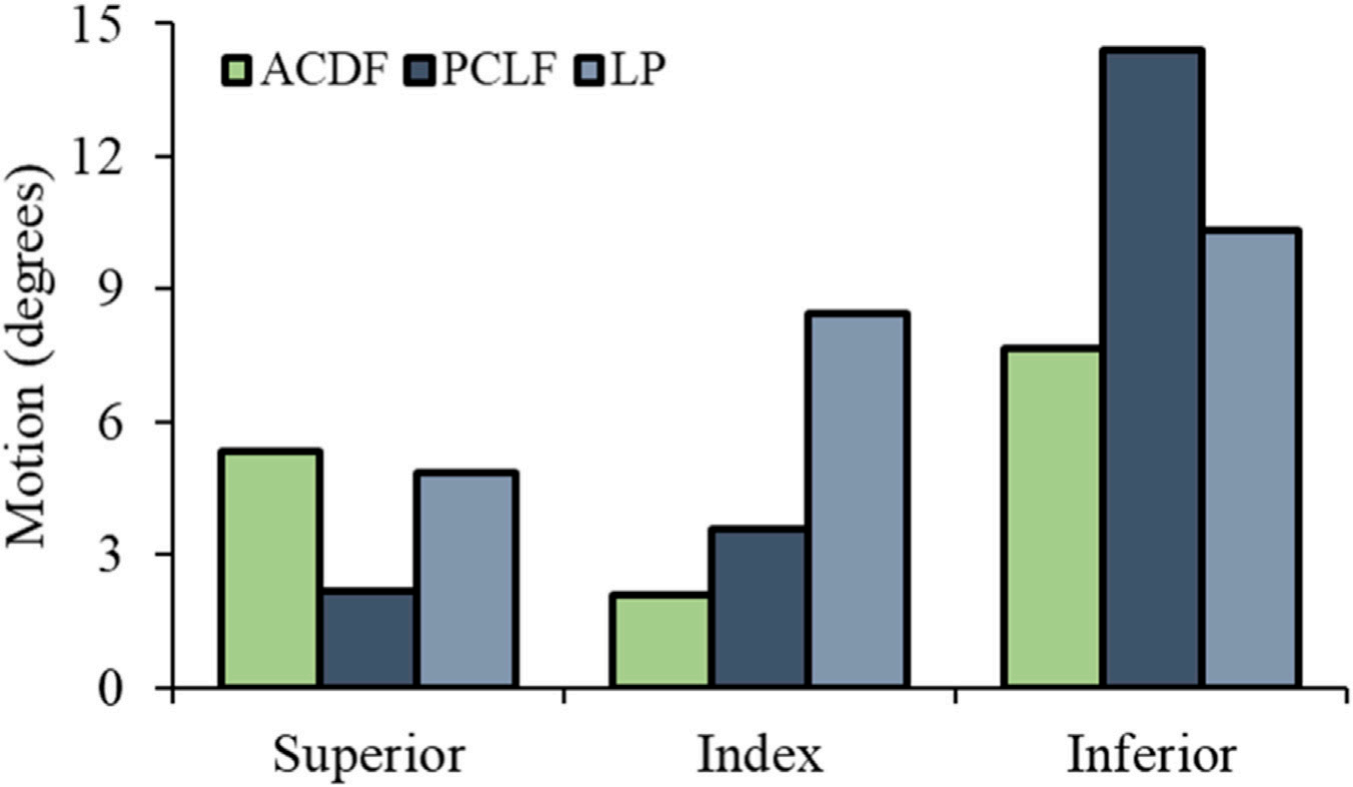
Cervical spine range of motions.

At the superior level, PCLF showed a slight ROM reduction (−8%) compared to ACDF’s significant decrease (−59%). LP exhibited a lesser ROM reduction. The index level observed a substantial increase in ROM for both PCLF (70%) and LP (302%) relative to ACDF, indicating increased mobility at the decompressed levels. At the inferior level, ROM increases were noted for PCLF (88%) and LP (35%) compared to ACDF (Figure 3). These findings suggest that PCLF and LP increases ROM, particularly at the index levels, with LP offering a substantial increases in ROM. PCLF and LP allow for increased ROM at the index levels, with LP showing a profound increase in ROM, thereby highlighting its potential for preserving motion post-surgery.

**FIGURE 4:**
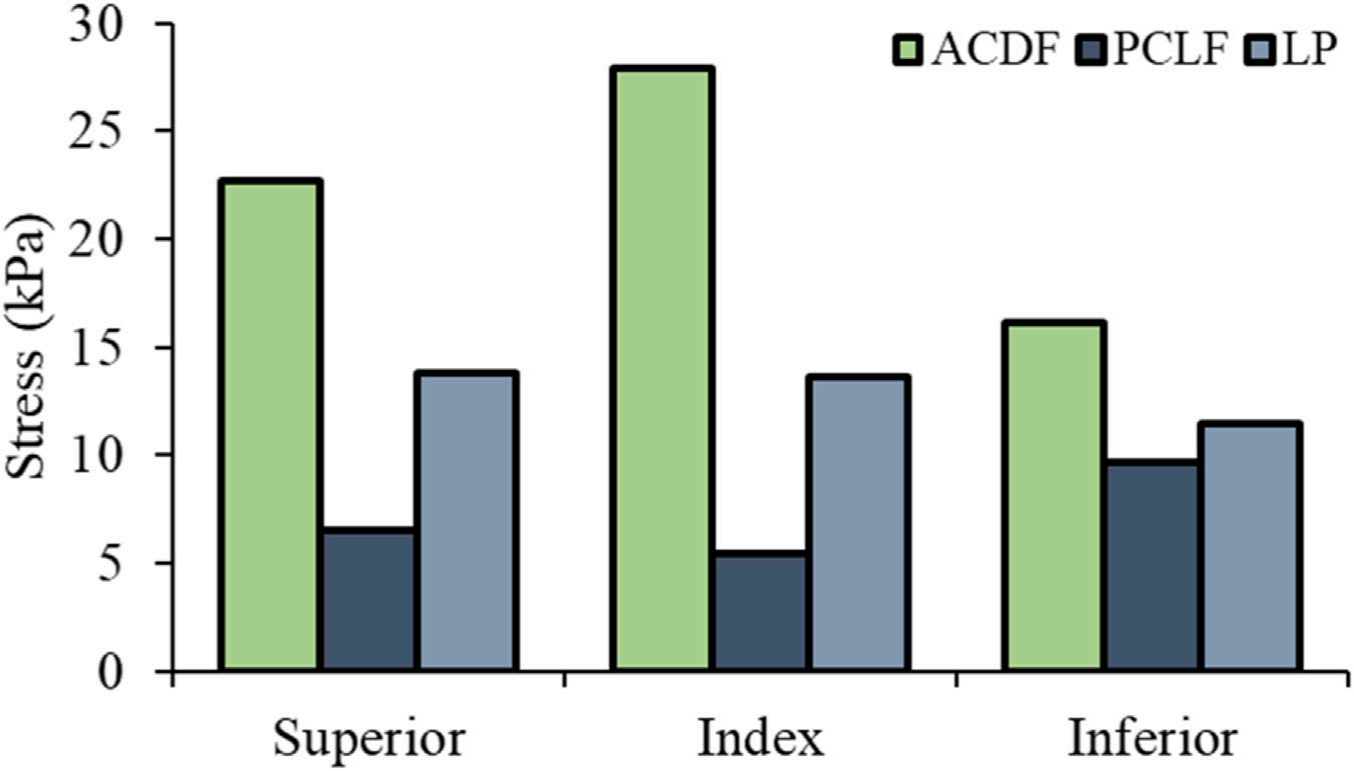
Spinal cord von Mises stress.

**FIGURE 5:**
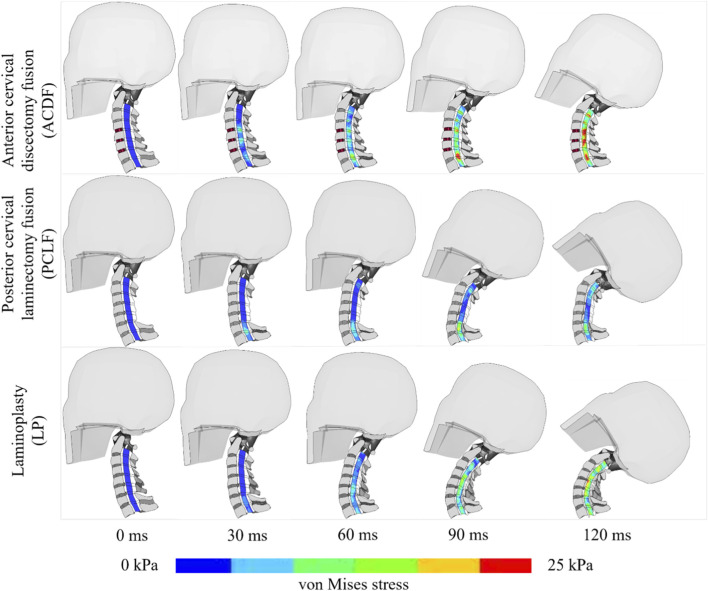
Spinal cord stress with different time intervals.

Superior Level: PCLF and LP showed a reduction in stress by −71% and −39% respectively compared to ACDF. Index Level: At the decompressed levels, there was a notable decrease in stress with PCLF (−81%) and LP (−51%) when compared with ACDF (Figure 4). Inferior Level: Both PCLF and LP exhibited lower stress reductions at −41% and −29% respectively. Figure 5 illustrates the spatial distribution of stress across the spinal cord in the mid-sagittal plane. When a peak scale value is set to 25 kPa, ACDF reaches peak stress in 90 ms, while LP reaches at 120 ms, and PCLF stays within the scale limit through out the event.

**FIGURE 6:**
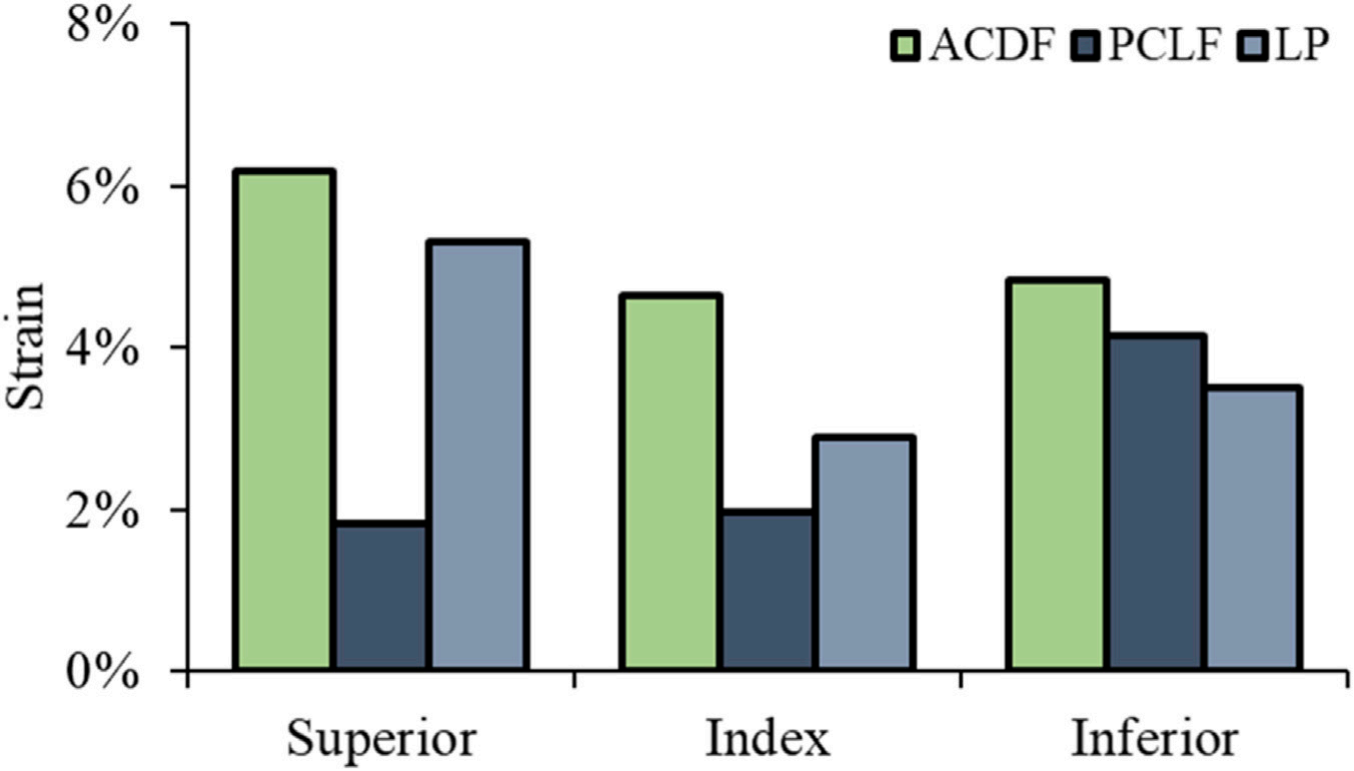
Spinal cord maximum principal strain.

**FIGURE 7:**
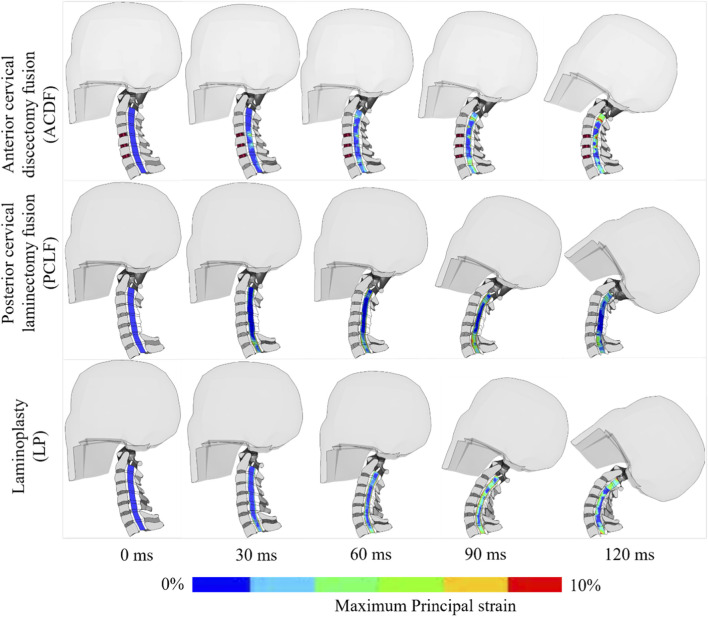
Spinal cord strain with different time intervals.

Superior Level: Strain reductions were −70% for PCLF and −14% for LP. Index Level: PCLF and LP demonstrated decreases in strains by −57% and −38%, respectively. Inferior Level: the reductions were −14% for PCLF and −27% for LP (Figure 6). The data indicate that both PCLF and LP are effective in reducing spinal cord stress and strain compared to ACDF, with PCLF showing more pronounced reductions in von Mises stress, particularly at the index and superior levels. Figure 7 illustrates the spatial distribution of the strain across the spinal cord in the mid-sagittal plane. The peak scale value is set to 10%. ACDF reaches peak strain at 90 ms, especially at adjacent levels, while LP reaches at 120 ms, and PCLF stays within the scale limit.

**FIGURE 8:**
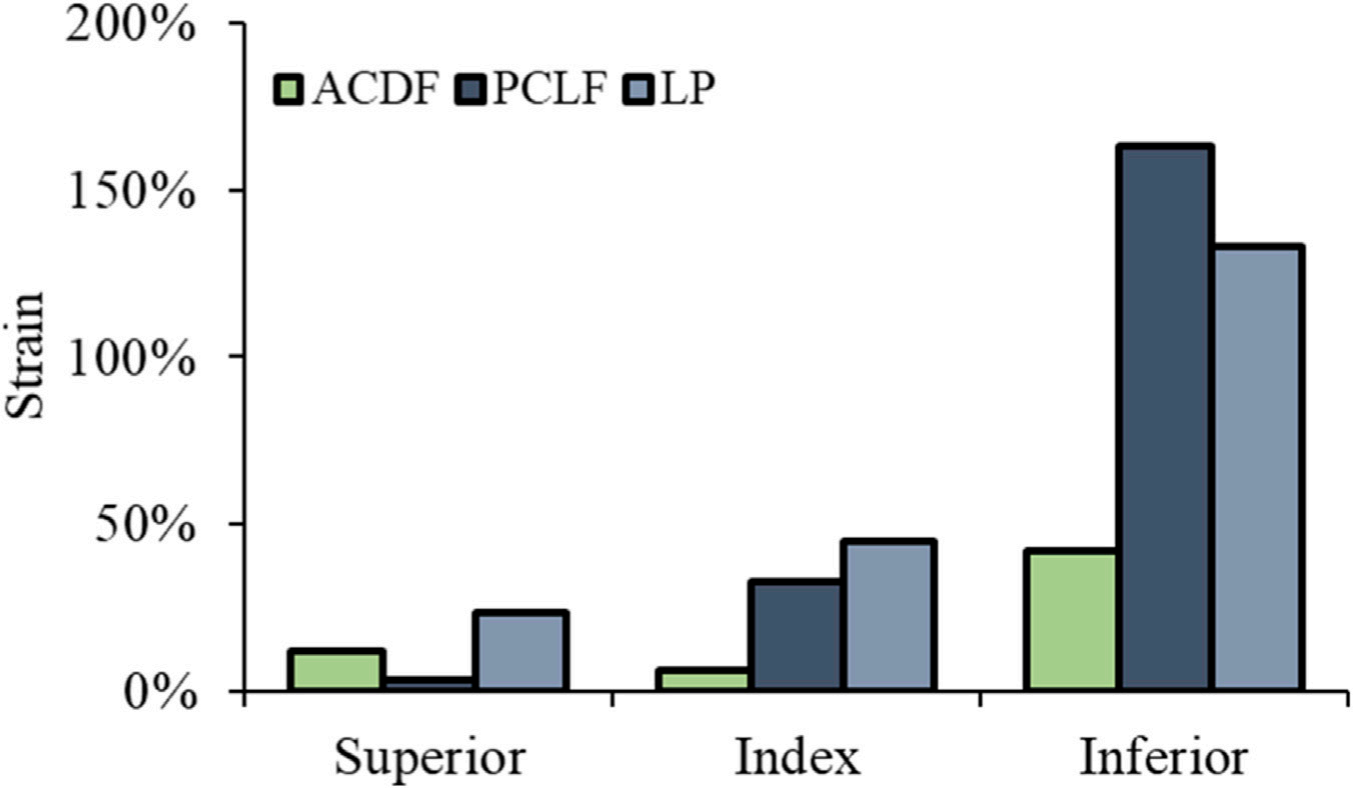
Capsular ligament strain.

Superior Level: CL strain decreased by 71% in PCLF but increased by 103% in LP. At the index level, CL strain increased to 4 times in PCLF and 6 times in LP. At the inferior adjacent level, both procedures resulted in 2 times increase in CL strain (Figure 8).

**FIGURE 9:**
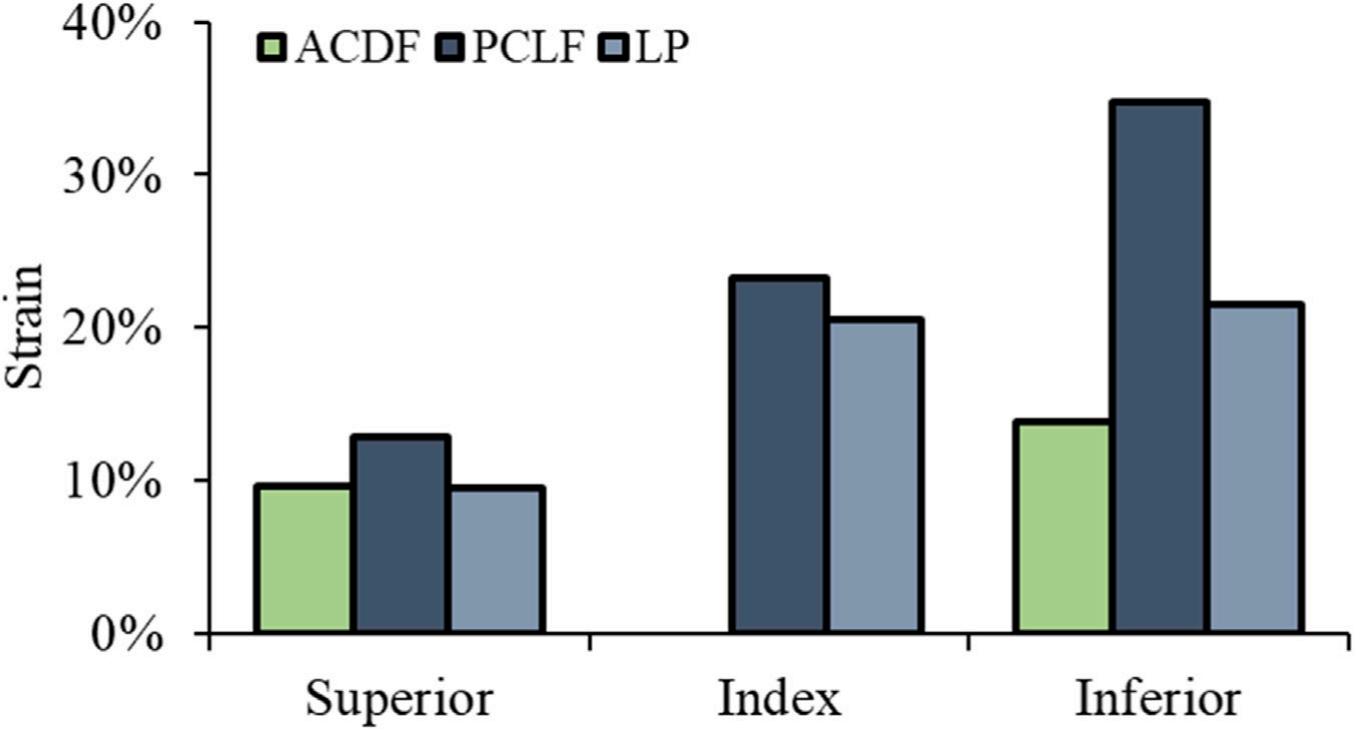
Anterior longitudinal ligament strain.

Superior Level: ALL strain slightly increased by 34% in PCLF and decreased by 1% in LP. Inferior Level: Substantial increases were seen, with PCLF showing a 151% increase and LP a 56% increase (Figure 9).

Instrument stress was evaluated in terms of peak von Mises stress. For ACDF, screw stresses ranged from 145 MPa at C6 to 398 MPa at C7. PCLF lateral mass screws displayed stresses from 210 MPa at C4 to 3,330 MPa at C3. In LP, lateral mass screw stresses were lower, from 99 MPa at C4 to 171 MPa at C6, and lamina screws showed 125 MPa at C5 to 251 MPa at C4. For plates/rods, the plate exhibited a peak stress of 587 MPa, the PCLF rod showed a much lower stress of 57 MPa, and the LP plates peaked at 527 MPa at C4.

## Discussion

In this study, we conducted simulations on three different cervical surgical interventions: ACDF, PCLF, and LP, simulated under the rear impact by applying a linear acceleration corresponding to a change in velocity of 2.6 m/s at T1 vertebrae, comparing their biomechanical outcomes with ACDF. The intact head-neck model was validated using range of motion data derived from human cadaver experiments for segments C2–C3 to C7–T1, ensuring the FE model’s response under load accurately reflected experimental data (Panjabi et al., 2004; Stemper et al., 2004). The rationale behind comparing between anterior approaches and posterior approaches, this comparison allows for an understanding of how different approaches alter spinal cord and column biomechanics under the rear impact.

For both fusion techniques, ACDF and PCLF, there was a substantial reduction in ROM at the index level. In contrast, LP, as a motion-preserving intervention, showed a higher ROM, as shown in cadaver studies (Huang et al., 2023). The ROM at the inferior level was higher than at the superior adjacent levels, consistent with previous in vivo and in vitro studies indicating hyperextension in the lower cervical spine during the initial phase of rear impact (John et al., 2019). The timing of the maximum S-curve, defined as the moment when C2–C3 reaches peak flexion, was observed at 40-45 ms across all surgical interventions, followed by an extension phase (John et al., 2019).

In rear impact scenarios characterized by extension movements, during extension the posterior elements of the spine are predominantly engaged. PCLF uses lateral mass screws and rods to effectively immobilize the spine, thus mitigating forces associated with such impacts (Graham et al., 1996; Lindsey and Miclau, 2000; Kiapour et al., 2022). In contrast, ACDF involves anterior fusion, which does not completely restrict extension movements, potentially increasing spinal cord stress and strain. While the range of motion is directly proportional to spinal cord stress and strain (Breig et al., 1966; Henderson et al., 2005). However, our study reveals that LP, despite allowing a higher range of motion than ACDF, results in minimal spinal cord stress and strain. This outcome is attributed to the posterior approach’s capacity to expand the fluid volume behind the spinal cord, acting as a buffer against impact forces (Ashana et al., 2021). These points demonstrate the biomechanical benefits of posterior surgical interventions in maintaining spinal integrity and reducing injury risk during rear impacts.

Muscle activation played a role, as evidenced by a study from Fice et al., which showed a reduction in CL strain from 28% to 13% with active muscles in a 7 g rear impact scenario (Fice et al., 2011). Study highlights the potential of predict soft tissue deformation during impacts, which could enhance our understanding of pain response mechanisms (Corrales and Cronin, 2021). Given that cervical facet joints are implicated as a primary source of chronic neck pain post-whiplash in 60% of cases (Lord et al., 1996). Previous cadaver studies have documented capsular ligament (CL) strains surpassing the 35% threshold, considered indicative of sub-traumatic failure, at load levels of 5 g and 10.5 g (Panjabi et al., 1998; Panjabi et al., 2004). In contrast, our investigation observed an average CL strain of 32.4% in PCLF procedures, which remains below this threshold. However, LP (LP) procedures at the index levels yielded a significantly higher average CL strain of 44.7%, thereby exceeding the sub-traumatic failure threshold of 35%. For the ALL, which is crucial in extension movements, the study by Yoganandan et al. 2000 suggests a strain threshold range of 40%-45% (Yoganandan et al., 2000). The average ALL strains were below this failure threshold, at under 36%, indicating a lower risk of damage. sOverall, posterior constructs reduced spinal cord stress and strain compared with ACDF during rear-impact loading. By increasing the posterior canal diameter, PCLF and LP permit dorsal spinal cord translation during extension-dominated motion, which reduces ventral contact and peak cord responses at the index levels. However, open-door laminoplasty produced the highest capsular ligament strains because the unilateral trough and hinge disrupt the posterior tension band and alter facet kinematics, leading to greater posterior translation, extension, and relative motion across the facets ligaments.

Instrument failure was evaluated by comparing the observed stress values to the yield strength of titanium alloy (Ti-6Al-4V), which is 900 MPa (Kulkarni et al., 2024; Tadepalli et al., 2011). In PCLF, lateral mass screws exhibited stress levels that surpassed the yield strength at all levels except C4, indicating a pronounced risk of failure for these screws under dynamic loads. However, the rod used in PCLF remained well within the safety limit. For ACDF and LP, the instruments exhibited stress levels below the yield threshold.

This study is limited by the use of low-speed rear impact simulations and lack of white and gray matter-specific tissue tension due to the nonavailability of human spinal cord white and gray matter tissue properties. Instrumentation was modeled as fully fused to bone using tied screw–bone and plate–bone interfaces, representing an ideal healed construct. This does not capture early postoperative states with micromotion which could increase motion and alter implant stresses and should be explored in future sensitivity analyses. Although we validated our FE model with experimental data, the interpretation of our results must consider the limitations of FE models for injury simulation. Our findings are based on a single male cervical spine model with neutral lordosis and average tissue properties, which limits generalizability. Variations in spinal curvature, vertebral geometry, muscle morphology, and sex-related anatomy may alter the quantitative response. Future work should use subject-specific models or parametric variations (e.g., different lordosis angles, bone density, and muscle cross-sectional area) to capture inter-individual variability. The outputs for the inferior adjacent segment may be unreliable due to the boundary condition effect, specifically the restriction of six degrees of freedom at the T1 vertebra. This study is limited to a single impact scenario (change in velocity 2.6 m/s) and assumes fully fused bone–implant interfaces. Future work should explore a range of impact speeds and directions, as well as partial bonding or screw loosening at the bone–implant interface (e.g., frictional or degraded contacts), to assess sensitivity to these factors and better represent early postoperative behavior. PCLF lateral mass screws experienced peak von Mises stresses above the assumed Ti-6Al-4V yield strength of 900 MPa. Screws were modeled as linear elastic and fully bonded to bone, so post-yield plasticity and fatigue were not represented; stresses above 900 MPa therefore mark regions where yielding would be expected rather than true elastic behavior beyond yield. This reduced safety margin for the PCLF hardware under extension-dominated rear-impact loading should be interpreted as a conservative indication of potential screw yielding or fracture, and future studies should incorporate elasto-plastic screw models and fatigue assessment to better quantify hardware-related risk. The neck position was neutral at the time of rear impact; however, neck rotation, flexion, or extension postures may alter spinal cord responses. True validation of the spinal cord responses is not feasible *in vivo*, and *in vitro*, cadaver studies will not be able to measure spinal cord stress or accurately recreate muscle activation. Global neck injury criteria commonly used in rear-impact assessment (e.g., NIC) were not computed, because the present analysis focused on segmental kinematics and tissue-level responses of the spinal cord and capsular ligaments. Despite these limitations, this is the first study to measure spinal cord and column responses in instrumented spine during rear impact.

## Conclusion

This study evaluated the biomechanical response of ACDF, PCLF, and LP under rear-impact loading using a validated finite element model. Compared to ACDF, posterior approaches reduced spinal cord stress and strain at the index level, with PCLF showing the greatest reduction despite similar reductions in segmental ROM for ACDF and PCLF. LP preserved the highest range of motion but resulted in elevated capsular ligament strain at the index level (44.7%) exceeding the sub-traumatic failure threshold of 35%, whereas PCLF remained below this threshold (32.4%); anterior longitudinal ligament strains remained below 36%, within the reported 40%–45% failure. Despite using a low-speed impact and long fusion, this is the first study to quantify spinal cord and implant responses in instrumented spines during rear impact. Clinically, these findings suggest that PCLF may be preferred when minimizing spinal cord loading under rear-impact conditions is a priority in rear impact scenario. These findings provide critical insights for surgical planning in trauma susceptible patients with prior cervical instrumentation.

## Data Availability

The original contributions presented in the study are included in the article/supplementary material, further inquiries can be directed to the corresponding author.
